# Associations of Serum Resistin With the Severity and Prognosis in Patients With Community-Acquired Pneumonia

**DOI:** 10.3389/fimmu.2021.703515

**Published:** 2021-11-09

**Authors:** Chun-Mei Feng, Jia-Yi Cheng, Zheng Xu, Hong-Yan Liu, De-Xiang Xu, Lin Fu, Hui Zhao

**Affiliations:** ^1^ Second Affiliated Hospital, Anhui Medical University, Hefei, China; ^2^ Department of Toxicology, Anhui Medical University, Hefei, China

**Keywords:** resistin, community-acquired pneumonia, inflammatory cytokines, severity, prognosis

## Abstract

**Background:**

Resistin is an endogenous ligand of Toll-like receptor 4 that activates several inflammatory signals. But the physiological function of resistin in community-acquired pneumonia (CAP) remains unknown. The goal of this research was to explore the associations between serum resistin and the severity and prognosis in CAP patients through a retrospective cohort study.

**Methods:**

All 212 CAP patients and 106 healthy cases were enrolled. Demographic characteristics were extracted. Serum resistin was determined *via* enzyme-linked immunosorbent assay. The prognosis was tracked in CAP patients.

**Results:**

Serum resistin on admission was raised in CAP patients compared with control cases. The level of resistin was gradually increased in parallel with CAP severity scores in CAP patients. Pearson and Spearman analyses revealed that serum resistin was positively correlated with CAP severity scores, white blood cells, urea nitrogen, creatinine, and inflammatory cytokines among CAP patients. There were negative relationships between resistin and hematocrit and albumin in CAP patients. Besides, linear and logistic regression analyses further indicated that serum resistin on admission was positively associated with CAP severity scores among CAP patients. Follow-up research revealed that serum resistin elevation on admission prolonged hospital stay in CAP patients.

**Conclusion:**

Serum resistin on admission is positively correlated with the severity and hospital stay in CAP patients, indicating that resistin may be involved in the physiological process of CAP. Serum resistin may be a potential biomarker in the diagnosis and prognosis for CAP.

## Introduction

Community-acquired pneumonia (CAP) is one of infectious diseases evoked by bacteria, viruses, or atypical pathogens. CAP brings tremendous danger to human health and elevates enormous strain on health-care resources all over the world. The frequencies of CAP diagnosed is 5~11/‰, and the mortality rate of adult hospitalized CAP patients is 5.7%~14.0% in the United States (1, 2). Due to the improvement of diagnostic techniques and therapeutic method, more etiology patterns of CAP have been distinguished in the past decades (3). However, the severity was inconsistent with the clinical manifestations of CAP patients under some circumstances. It is difficult to timely care for CAP patients because of insufficient sample collection, delayed process, and antimicrobial therapy (4). Therefore, it is beneficial to develop a rapid diagnostic method or search for a new biomarker for alleviating the severity and mortality in CAP patients.

Obesity is associated with several comorbidities, such as diabetes and hypertension, as well as higher mortality because of elevated body mass index (BMI) (5, 6). Recently, several studies suggested that obesity is a risk factor of infectious diseases such as CAP, influenza, and coronavirus disease 2019 (7–9). Resistin is firstly discerned in the white adipocytes of murine animals and is a member of cysteine-rich protein family (10). The expression pattern of resistin is significantly different between rodents and humans. Resistin is mainly expressed in white adipose tissue of murine animals and in monocytes, macrophages, and adipocytes of human beings (11). Therefore, resistin could lead to insulin resistance and inflammatory reaction. A few studies indicated that resistin was increased in many inflammatory diseases (12, 13). Several reports from our team have revealed that many indices of inflammation and oxidative stress are increased in patients with CAP (14, 15). Consequently, it is reasonable to speculate that resistin may be involved in CAP. However, the exact role of resistin was unclear among CAP patients.

Given that obesity is related to elevating the risks of death and severity in CAP patients, it could be assumed that resistin may participate in the pathophysiological process of CAP. But only few clinical data illuminated the association of resistin with CAP. Therefore, serum resistin was measured in CAP patients. The correlations between serum resistin and severity and prognosis were investigated in CAP patients based on a retrospective cohort study.

## Materials and Methods

### Subjects

This research was a retrospective cohort study. All participators were selected from the Respiratory and Critical Care Medicine of Second Affiliated Hospital in Anhui Medical University from April 2018 to April 2020. In total, 212 CAP patients (119 males and 93 females) were recruited in this study. Demographic characteristics and clinical information were collected. CAP referred to the inflammation of infectious pulmonary parenchyma (including alveolar wall and interstitial lung in a broad sense) outside the hospital, including pneumonia with a definite incubation period. Clinical diagnosis of CAP must meet the diagnostic criteria (16). Pneumonia severity was evaluated by CAP severity scores, including Pneumonia Severity Index (PSI), CURB-65, CRB-65, CURXO, and SMART-COP scores (17). The inclusion criteria were as follows: patients were more than 18 years old; occurred in a patient who was not hospitalized or residing in a long-term care facility for more than 2 weeks before the onset of symptoms; patients were not admitted to the hospital in the last 3 months; did not reside in nursing homes; were not pregnant and had no pulmonary malignant tumor; had no treatment or intervention before hospitalization. Moreover, age-, sex-, and BMI-matched healthy subjects were selected and enrolled from the physical examination center in the Second Affiliated Hospital of Anhui Medical University. Through electronic medical records, control subjects with comorbidities were eliminated in the current research. Fasting blood samples were collected at the same time of day among CAP patients and healthy subjects after having received written informed consent. The night before blood was drawn, all participators did not eat or drink after 12:00 a.m. When CAP patients have made a good recovery, fasting blood samples were again collected before CAP patients were discharged from the hospital. This study was approved by the ethics committee of Anhui Medical University.

### Enzyme-Linked Immunosorbent Assay

Serum fasting samples were drawn from participants in the morning and centrifugated at a speed of 3,000 rpm. Resistin and circulating inflammatory cytokines were measured in serum through ELISA. Resistin ELISA kits were purchased from Cusabio, Wuhan, China (https://www.cusabio.com/). Inflammatory cytokine ELISA kits were obtained from Wuhan ColorfulGene Biological Technology Co., Ltd. (http://www.jymbio.com/). All detections were conducted in accordance with the previous study (18).

### Statistical Analysis

Statistical analyses were carried out with SPSS 18.0 software. Categorical variables were shown as counts (percentages). Continuous variables were expressed as mean for normally distributed data and median for skewed data. All categorical variables were compared by the Fisher’s exact test or χ^2^ test. The difference between CAP patients and control subjects was analyzed *via* Student’s *t*-test or nonparametric test. The associations of serum resistin with inflammatory cytokines and the clinical indices of CAP were explored through Spearman and Pearson correlation analyses. Then, the date of CURB-65, CRB-65, PSI, and SMART-COP were normally transformed, and the correlations between serum resistin and CAP severity scores were further investigated through linear and logistic regression analyses. The association between serum resistin and the prognosis was evaluated using logistic regression. *P* value was two-sided, and statistical significance was determined at *P* ≤ 0.05.

## Results

### Demographic Characteristics and Clinical Information

All 212 patients with CAP and 106 control cases were included in this study. The demographic characteristics and clinical information were analyzed. As expressed in **Table 1**, there was no difference in gender, age, BMI, and systolic and diastolic pressures between the two groups. The blood routine indices were measured on admission. The results indicated that the counts of white blood cells (WBCs) and neutrophils were elevated in patients with CAP. On the contrary, the number of lymphocytes was decreased in CAP patients compared with control cases. Moreover, the ratios of platelet to lymphocyte (PLR), neutrophil to lymphocyte (NLR), and monocyte to lymphocyte (MON) were increased in CAP patients (**Table 1**). In addition, we found that the levels of cholesterol and triglyceride were slightly reduced in CAP patients than those in control subjects. Glucose was similar between the two groups (**Table 1**). Additionally, urea nitrogen was elevated and uric acid was dropped in CAP patients. There was no difference in alanine aminotransferase (ALT) and aspartate aminotransferase (AST) between the two groups. Besides, pro-inflammatory cytokines including tumor necrosis factor (TNF)-α, interleukin (IL)-1β, IL-6, and C-reactive protein (CRP) were elevated in CAP patients (**Table 1**).

**Table 1:** Demographic and biochemical characteristics between CAP patients and control subjects.

Variables	CAP (n = 212)	Control (n = 106)	P
Male, n (%)	119 (56.1)	56 (52.8)	0.586
Age (years)	66.2 (51.5, 80.2)	62.3 (48.5, 78.5)	0.452
BMI	22.4±0.46	21.6±0.75	0.221
Systolic pressure (mmHg)	121.1±2.13	114.6±4.51	0.564
Diastolic pressure (mmHg)	72.3±1.11	75.6±2.36	0.521
WBC (10^9^/L)	6.90 (5.04, 9.35)	5.73 (4.66, 6.65)	<0.05
Neutrophil (10^9^/L)	4.97 (3.10, 7.43)	3.04 (2.39, 3.88)	<0.01
Lymphocyte (10^9^/L)	1.23 (0.82, 1.85)	2.11 (1.80, 2.43)	<0.01
NLR	3.92 (2.13, 9.01)	1.44 (1.24, 2.21)	<0.01
MON	0.35 (0.22, 0.58)	0.17 (0.15, 0.22)	<0.05
PLR	182.5 (119.3, 340.8)	106.1 (87.4, 133.7)	<0.01
Glucose (mmol/L)	5.4±0.12	5.0±0.05	0.452
Cholesterol (mmol/L)	4.1±0.12	4.9±0.07	<0.05
Triglyceride (mmol/L)	1.0±0.07	1.6±0.09	<0.05
ALT (U/L)	27.6±2.62	25.1±1.38	0.698
AST (U/L)	30.6±2.15	25.6±1.57	0.345
Urea nitrogen (mmol/L)	6.3±0.16	4.5±0.09	<0.05
Creatinine (μmol/L)	68.2±0.35	62.5±0.14	0.105
Uric acid (μmol/L)	275.6±1.35	368.2±8.97	<0.05
TNF-α (pg/mL)	561.0 (292.8, 1121.8)	66.5 (40.3, 95.3)	<0.01
IL-1β (pg/mL)	361.6 (197.2, 581.2)	56.9 (20.3, 82.3)	<0.01
IL-6 (pg/mL)	70.6 (43.6, 94.3)	38.5 (20.5, 58.6)	<0.01
CRP (mg/L)	44.2 (5.2, 98.2)	10.3 (2.5, 28.6)	<0.01
CURB-65	2.0 (0, 3.0)	N.A	N.A
CRB-65	1.0 (0, 2.0)	N.A	N.A
PSI	98.0 (59.0, 131.0)	N.A	N.A
CURXO [Severe, n (%)]	90 (42.5)	N.A	N.A
SMART-COP	2.0 (0, 5.0)	N.A	N.A

Categorical variables were shown as counts (percentages). Continuous variables were expressed as mean for normally distributed data and median for skewed data. All categorical variables were compared by the Fisher exact test or χ^2^ test. The difference of normally distributed data was compared using student's t test between CAP patients and control subjects. The difference of abnormally distributed data was compared through Mann-Whitney U test between two groups. N.A., Not available.

### The Levels of Serum Resistin Between Control Cases and Community-Acquired Pneumonia Patients

**Figure 1:**
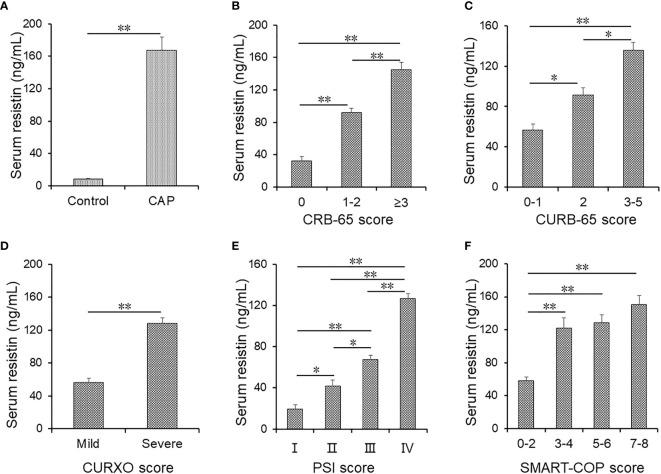
The levels of serum resistin in community-acquired pneumonia (CAP) patients and control cases. **(A–F)** Serum resistin was detected using ELISA. **(A)** Serum resistin in CAP patients and control subjects. **(B)** Serum resistin in different CRB-65 scores in CAP patients. **(C)** Serum resistin in different CURB-65 scores in CAP patients. **(D)** Serum resistin in different CURXO scores in CAP patients. **(E)** Serum resistin in different Pneumonia Severity Index (PSI) scores in CAP patients. **(F)** Serum resistin in different SMART-COP scores in CAP patients. All data were expressed as mean ± SEM. *P < 0.05, **P < 0.01.

Resistin was detected in serum between control cases and CAP patients. The level of resistin was increased in CAP patients (**Figure 1A**). The levels of serum resistin were further compared in the different grades of CAP patients. The results found that serum resistin was lower in grade 0 than those in grades 1–2 and ≥3 in accordance with CRB-65 score (**Figure 1B**). Parallel with the CURB-65 score, serum resistin was gradually elevated (**Figure 1C**). Moreover, serum resistin was measured in mild and severe patients on the basis of CURXO score. As shown in **Figure 1D**, serum resistin was raised in the severe patients with CAP. In addition, serum resistin was gradually risen in line with PSI score among CAP patients (**Figure 1E**). Finally, the levels of serum resistin were compared depending on SMART-COP score; the level of serum resistin was lower in patients with 0–2 scores than those in other grades (**Figure 1F**).

### Correlations of Serum Resistin With Clinical Characteristics in Community-Acquired Pneumonia Patients

The correlations of serum resistin with clinical markers were analyzed using Spearman and Pearson rank correlation in CAP patients. Serum resistin was positively associated with WBCs (*r* = 0.266; *P* < 0.05) and neutrophils (*r* = 0.239; *P* = 0.001) and inversely correlated with lymphocytes (*r* = *-*0.421; *P* < 0.001) and hematocrit (*r* = 0.417; *P* < 0.001) in CAP patients (**Table 2**). Moreover, the relationship of serum resistin with renal function and liver function was analyzed. As shown in **Table 2**, the results indicated that serum resistin was positively associated with urea nitrogen (*r* = 0.417; *P* < 0.001) and creatinine (*r* = 0.270; *P* = 0.007). Besides, there was a negative correlation between serum resistin and albumin (*r* = -0.601; *P* < 0.001). In addition, serum resistin was dramatically and positively correlated with TNF-α (*r* = 0.631; *P* < 0.001), IL-1β (*r* = 0.798; *P* < 0.001), and CRP (*r* = 0.383; *P* = 0.001). Statistically nonsignificant difference between serum resistin and IL-6 was found in CAP patients.

**Table 2:** Correlations between serum resistin with the severity, blood routine parameters, liver function, renal function and inflammatory cytokines in CAP patients.

Variables	CURB-65	CRB-65	PSI	CURXO	SMART-COP
***r***	0.719	0.734	0.625	0.456	0.548
***P***	<0.001	<0.001	<0.001	<0.001	<0.001
**Variables**	WBC	Neutrophil	Lymphocyte	Hematocrit	Urea nitrogen
***r***	0.266	0.329	-0.488	-0.421	0.417
***P***	0.008	0.001	<0.001	<0.001	<0.001
**Variables**	Creatinine	Uric acid	ALT	AST	TBIL
***r***	0.270	-0.156	-0.196	-0.048	-0.012
***P***	0.007	0.124	0.053	0.636	0.904
**Variables**	Albumin	TNF-α	IL-1β	CRP	IL-6
***r***	-0.601	0.631	0.798	0.383	0.037
***P***	<0.001	<0.001	<0.001	0.001	0.793

The associations of serum resistin with CAP severity scores, blood routine parameters and inflammatory cytokines were accessed through Spearman correlation analysis. The associations of serum with the indices of liver function and renal function were evaluated through Pearson correlation analysis.

### Associations of Serum Resistin With the Severity in Community-Acquired Pneumonia Patients

The correlations of serum resistin with CAP severity scores were analyzed in CAP patients using Pearson rank correlation. Serum resistin was significantly and positively correlated with CURB-65 (*r* = 0.719; *P* < 0.001), CRB-65 (*r* = 0.734; *P* < 0.001), PSI (*r* = 0.625; *P* < 0.001), CURXO (*r* = 0.456; *P* < 0.001), and SMART-COP (*r* = 0.548; *P* < 0.001) (**Table 2**). Simultaneously, the association between serum resistin and the severity was further explored *via* linear regression analysis and logistic regression analysis among CAP patients. As shown in **Table 3**, univariable linear regression analysis revealed that serum resistin was positively associated with CURB-65 score (β: 0.515; 95% CI: 0.002~0.714), CRB-65 score (β: 0.542; 95% CI: 0.015~0.721), PSI score (β: 0.354; 95% CI: 0.112~0.765), and SMART-COP (β: 0.448; 95% CI: 0.033~0.234) among CAP patients. Univariable logistic regression analysis found that serum resistin was positively associated with CURXO score (OR: 1.189; 95% CI: 1.002~1.456) in CAP patients. In order to control confounding factors, age, sex, BMI, and comorbidities were adjusted. Multivariable linear regression analysis revealed serum resistin was positively associated with CURB-65 score (β: 0.472; 95% CI: 0.005~0.019), CRB-65 score (β: 0.497; 95% CI: 0.004~0.654), PSI score (β: 0.320; 95% CI: 0.092~0.917), and SMART-COP (β: 0.312; 95% CI: 0.026~0.051) in CAP patients. Multivariable logistic regression analysis found that serum resistin was positively associated with CURXO score (OR: 1.160; 95% CI: 1.016~1.306) in CAP patients (**Table 3**).

**Table 3:** Associations between serum resistin with CAP severity scores among CAP patients.

Variables	Univariable (β, 95% CI)	P	Multivariable (β, 95% CI)*	P
CURB-65	0.515 (0.002, 0.714)	<0.001	0.472 (0.005, 0.019)	0.001
CRB-65	0.542 (0.015, 0.721)	<0.001	0.497 (0.004, 0.654)	0.001
PSI	0.354 (0.112, 0.765)	<0.001	0.320 (0.092, 0.917)	0.011
SMART-COP	0.448 (0.033, 0.234)	0.032	0.312 (0.026, 0.051)	0.046
	Univariable (OR, 95% CI)	*P*	Multivariable (OR, 95% CI)*	*P*
CURXO	1.189 (1.002, 1.456)	0.021	1.160 (1.016, 1.306)	0.007

*Adjusted for age, sex, BMI and comorbidities.

The date of CURB-65, CRB-65, PSI and SMART-COP were normally transformed. Then, the associations of serum resistin with CURB-65, CRB-65, PSI and SMART-COP were determined through linear regression analysis. The association of serum resistin with CURXO was analyzed via logistical regression analysis.

### Receiver Operating Characteristic Curves and Cutoff Point Analysis for Serum Resistin

**Figure 2:**
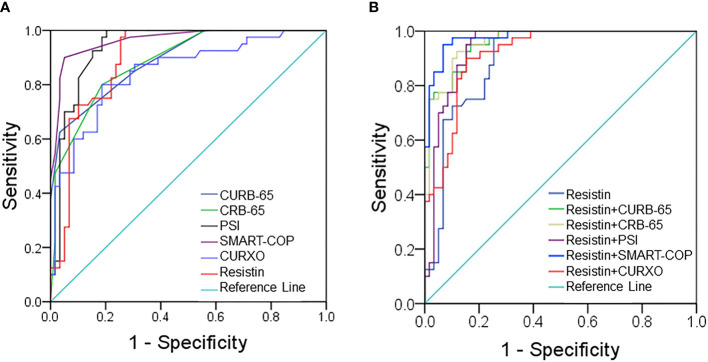
Receiver operating characteristic (ROC) curves for different predictive indexes on admission. **(A)** ROC curve was used to evaluate the predictive values of serum resistin and community-acquired pneumonia (CAP) severity scores for CAP. **(B)** ROC curve was used to evaluate the predictive values of serum resistin combination with CAP severity scores for severity.

Moreover, the predictive powers of resistin and CAP severity scores for severity were analyzed using receiver operating characteristic (ROC) area under the curve (AUC) in CAP patients. As shown in **Figure 2A**, the AUC of serum resistin for CAP was 0.893 (95% CI: 0.832, 0.954). The sensitivity and specificity of high resistin concentrations were 76.8% and 87.6%, respectively. Moreover, the AUCs for the severity were evaluated in CAP patients. As shown in **Figure 2A**, the AUCs were as follows: resistin, 0.893 (95% CI: 0.832, 0.954); CURB-65, 0.886 (95% CI: 0.823, 0.950); CRB-65, 0.939 (95% CI: 0.891, 0.987); PSI, 0.965 (95% CI: 0.932, 0.998); SMART-COP, 0.901 (95% CI: 0.768, 0.941); and CURXO, 0.896 (95% CI: 0.834, 0.959). The optimal cutoff value of resistin was 111.56 ng/ml, with 79.0% sensitivity and 81.0% specificity. Moreover, the predictive powers of resistin combination with CAP severity scores for severity were evaluated among CAP patients. As shown in **Figure 2B**, the AUCs were as follows: Resistin, 0.893 (95% CI: 0.832, 0.954); Resistin+CURB-65, 0.959 (95% CI: 0.926, 0.992); Resistin+CRB-65, 0.961 (95% CI: 0.929, 0.994); Resistin+PSI, 0.940 (95% CI: 0.893, 0.988); Resistin+SMART-COP, 0.978 (95% CI: 0.954, 1.001); and Resistin+CURXO, 0.917 (95% CI: 0.865, 0.970).

### The Association Between Serum Resistin and the Prognosis in Community-Acquired Pneumonia Patients

**Figure 3:**
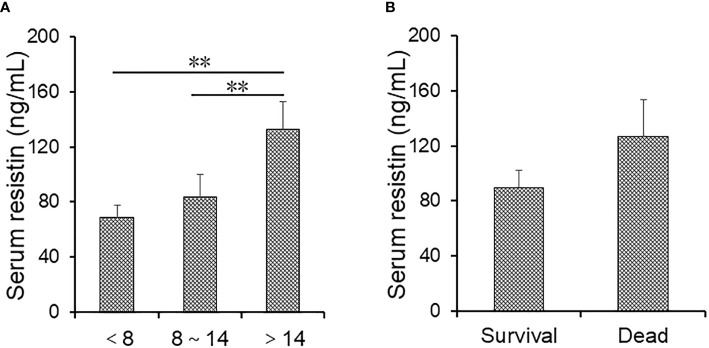
The levels of serum resistin in community-acquired pneumonia (CAP) patients with different prognostic outcomes. **(A, B)** Serum resistin was measured through ELISA. **(A)** The levels of serum resistin in alive CAP patients with different hospital stays. **(B)** The levels of serum resistin in alive cases and dead patients. All data were expressed as mean ± SEM. **P < 0.01.

Serum resistin was compared in CAP patients with different hospital stays. As shown in **Figure 3A**, serum resistin was higher in patients over 14 days than those below 8 days and from 8 to 14 days. Moreover, there was no difference in resistin between survival cases and dead patients (**Figure 3B**). Furthermore, the association between serum resistin and the prognosis was explored using logistic regression analysis among CAP patients. The univariate logistic regression analysis indicated that serum resistin on admission was positively associated with hospital stay over 14 days (OR = 1.316; 95% CI: 1.102~1.653) (**Table 4**). Further multivariate logistical regression found that serum resistin elevation obviously prolonged the hospital stay over 14 days by 1.268 (OR = 1.268; 95% CI: 1.112~1.563) (**Table 4**). In addition, the levels of serum resistin were further compared in CAP patients before treatment and after treatment. As shown in **Supplementary Figure S1**, the level of serum resistin was decreased in CAP patients after treatment compared with those in CAP patients before treatment.

**Table 4:** Association between serum resistin and hospital stay among CAP patients.

Hospital stays	Univariate (OR, 95% CI)	P	Multivariate (OR, 95% CI)*	P
<8	1	*_*	1	*_*
8~14	1.132 (0.965, 1.215)	0.235	1.326 (0.924, 1.461)	0.562
>14	1.316 (1.102, 1.653)	0.023	1.268 (1.112, 1.563)	0.047

*Adjusted for age, sex, BMI and comorbidities.

The association of serum resistin and hospital stay was analyzed via logistical regression analysis.

## Discussion

From what has been discussed above, the present project was the first epidemiological study to analyze the relationship of serum resistin with the severity and prognosis in CAP patients based on a large retrospective cohort study conducted in a tertiary teaching hospital. These results of this study mainly find that serum resistin was increased in CAP patients, serum resistin was gradually elevated consistent with CAP severity scores in CAP patients, serum resistin was positively correlated with CAP severity scores among CAP patients, and serum resistin elevation prolonged hospital stays among CAP patients.

Previous studies found that resistin may be regarded as an inflammatory cytokine associated with a number of inflammatory diseases (12, 13). Serum resistin was elevated in the patients with chronic obstructive pulmonary disease after *Chlamydia pneumoniae* infection (19). Moreover, the level of hepatic resistin was increased in mice exposed to *Escherichia coli* (20). Nevertheless, there were few epidemiological studies investigating the function of resistin in CAP patients. In this study, serum resistin was detected in CAP patients and control subjects. We found that serum resistin was significantly increased in CAP patients. Besides, the elevation of serum resistin paralleled CAP severity scores among CAP patients. Correlative analysis indicated that serum resistin was positively correlated with CAP severity scores. In order to further analyze the association between serum resistin and the severity of CAP, linear and logistic regression analyses were conducted. These results suggested that serum resistin elevation was an independent risk factor of the growing severity of CAP patients. Moreover, a follow-up study was conducted. We found that serum resistin was positively associated with hospital stay in CAP patients. Not only that, the symptoms of pneumonia were alleviated and the level of resistin was decreased in CAP patients after treatment. Generally speaking, these results provide evidence that serum resistin is positively associated with the severity and hospital stay in CAP patients.

Mounting evidence has suggested that inflammatory cytokines are involved in the occurrence and development of CAP. Many circulating inflammatory cytokines were elevated in CAP patients. Not only that, inflammatory cytokines were positively related with the severity of CAP (21–25). Moreover, an animal experiment found that resistin enhanced inflammatory cytokine production through activating the nuclear factor (NF)-κB signal in coronary artery tissues of mice (26). However, the correlation of serum resistin with inflammatory cytokines remained obscure among CAP patients. In this study, the relationships between serum resistin and different inflammatory cytokines were explored in CAP patients. The results found that there were positive correlations between serum resistin and inflammatory cytokines among CAP patients. Furthermore, an earlier project from our research group found that blood routine indices, liver function, and renal function can serve as indicators for pneumonia (27–29). In this issue, we found that serum resistin was positively associated with WBCs, neutrophil, urea nitrogen, and creatinine and negatively associated with lymphocyte, hematocrit, and albumin among CAP patients. In addition, the predictive powers of serum resistin and CAP severity scores for severity were counted using ROC curve test. We found that the predictive capacity of serum resistin was similar with CAP severity scores. Meanwhile, the predictive capacity of serum resistin combination with CAP severity scores for severity was evaluated through ROC curve test. The data indicated that serum resistin combination with CAP severity scores elevated the predictive capacity for severity than single serum resistin and CAP severity scores among CAP patients. These results demonstrate that resistin may be regarded as a potential biomarker for diagnosis and prognosis in CAP.

Resistin is an adipokine, which is mainly expressed in white adipose tissue of murine animals and in monocytes, macrophages, and adipocytes of human beings (11). Therefore, resistin could contribute to insulin resistance and inflammatory reaction. The role of resistin in the CAP is scarcely clear. Resistin has been known as a protein “found in the inflammatory zone” (FIZZ). Past studies indicated that resistin was evidently elevated in patients with inflammatory diseases (12, 13, 21). Moreover, some pro-inflammatory cytokines, such as TNF-α, IL-6, and lipopolysaccharide (LPS) can regulate the level of resistin mRNA. TNF-α exposure obviously elevated resistin mRNA in human peripheral blood mononuclear cells (30). In addition, adipocytes are immune cells. Inflammation can activate immune cells and evoke an immune response in human bodies (31). Resistin is the downstream of the immune response, and both amplify the inflammatory response and mediate some of its effects (32). Moreover, recent studies reported that resistin bound to an isoform of decorin and exerted important effects on cellular proliferation and migration (33). Another study revealed that human resistin could bind to Toll-like receptor 4 (TLR4), the innate receptor for LPS, and activate inflammatory signal pathways (34). Human resistin mimicked the function of LPS, competed with LPS for binding to TLR4 and then activated several inflammatory signal pathways, such as NF-κB, signal transducer and activator of transcription (STAT)-3, and mitogen-activated protein kinases (MAPKs), and finally mediated the inflammatory response (35–38). Resistin induced the secretion of pro-inflammatory cytokines and chemokines, such as IL-1β, macrophage inflammatory protein (MIP)-2, IL-6, and TNF-α (36, 39). Therefore, we speculate that pathogen infection evokes inflammation elevation in CAP patients. Inflammatory cytokines not only directly increase resistin but also activate the immune response in adipocytes and elevate the downstream resistin. In turn, resistin elevation further aggravates inflammatory activation and the secretion of inflammatory cytokines among CAP patients. Our study also found that inflammatory cytokines were increased in CAP patients. Therefore, this evidence indicates that resistin may be involved in the process of CAP partially through directly activating inflammatory signal pathways and secreting circulating inflammatory cytokines.

There were several defects in this study that need to be considered. First, the sample size was relatively modest and all the subjects were from a single center. Further studies with larger sample sizes from multicenters are needed to verify these results. Second, the mechanism leading to resistin elevation in patients with CAP was not unclear. More laboratory research is needed next. Third, the level of systemic resistin was measured in serum; the level of local resistin was uncharted. Therefore, we will test the level of resistin in bronchoalveolar lavage fluid. Fourth, it was an epidemiological study, so the causal relationship was obscure. Further *in vivo* and *in vitro* experiments are needed to confirm these results in the future.

## Conclusions

The present study has some strengths. It is the first epidemiological study to analyze the association of serum resistin with the severity and prognosis among CAP patients. These results found that serum resistin is elevated in CAP patients. Serum resistin is gradually elevated in parallel with the severity of CAP patients. Serum resistin is positively associated with the severity and poor prognosis among CAP patients. Therefore, we suggest that serum resistin detection can be beneficial for making decisions in CAP clinical management. Serum resistin may be used to predict the severity and prognosis as a potential biomarker for CAP.

## Data Availability Statement

The original contributions presented in the study are included in the article/**Supplementary Material**. Further inquiries can be directed to the corresponding authors.

## Ethics Statement

The studies involving human participants were reviewed and approved by the ethics committee of Anhui Medical University. The patients/participants provided their written informed consent to participate in this study.

## Author Contributions

LF, HZ, and D-XX conceived the study. LF designed the study. LF, HZ, D-XX, C-MF, H-YL, J-YC, and ZX performed the research. LF conducted statistical analyses of all data. LF drafted the article. All authors contributed to the article and approved the submitted version.

## Funding

This study was supported by the National Natural Science Foundation of China (82100078 and 81670060), National Natural Science Foundation Incubation Program of the Second Affiliated Hospital of Anhui Medical University (2020GQFY05), and Scientific Research of Health Commission in Anhui Province (AHWJ2021b091).

## Conflict of Interest

The authors declare that the research was conducted in the absence of any commercial or financial relationships that could be construed as a potential conflict of interest.

## Publisher’s Note

All claims expressed in this article are solely those of the authors and do not necessarily represent those of their affiliated organizations, or those of the publisher, the editors and the reviewers. Any product that may be evaluated in this article, or claim that may be made by its manufacturer, is not guaranteed or endorsed by the publisher.
