# Supplementary material for: Associations of Serum Resistin With the Severity and Prognosis in Patients With Community-Acquired Pneumonia
Source: Front Immunol. 2021 Nov 9;12:703515. doi: 10.3389/fimmu.2021.703515 (PMC8630736; doi:10.3389/fimmu.2021.703515)
Supplement: Supplementary file 1 [file DataSheet_1.docx]

**Supplementary Data**


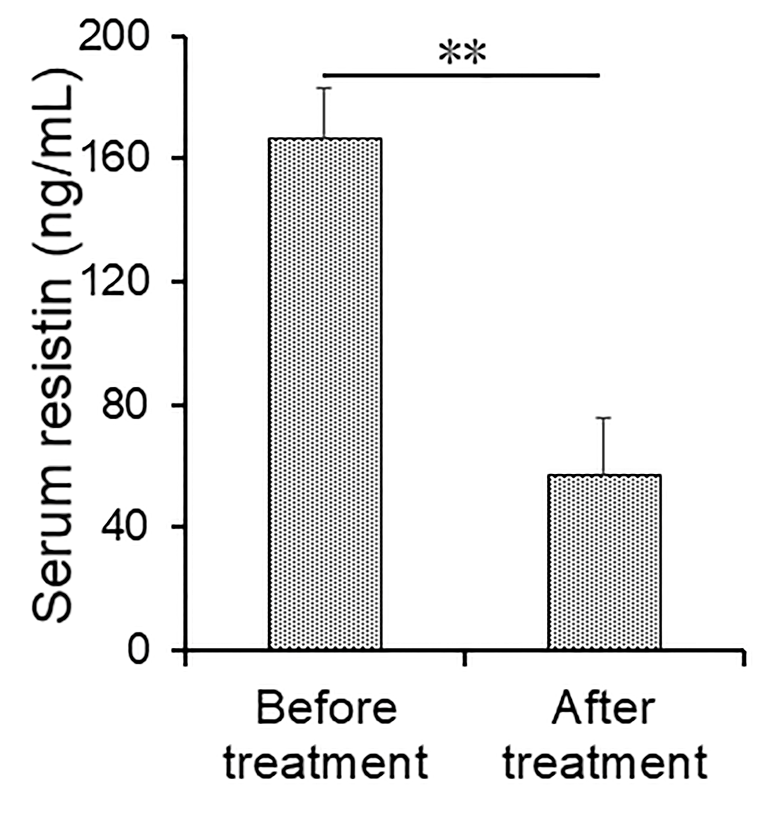


**Supplemental Figure 1. The levels of serum resistin in CAP patients between before treatment and after treatment.** When CAP patients have made a good recovery, fasting blood samples were again collected before CAP patients were discharged from hospital. The levels of serum resistin were detected and compared in CAP patients between before treatment and after treatment. All data were expressed as mean ± SEM. ***P*＜0.01.
